# The Impact of a Personal Cancer Diagnosis on the Psychological Health of Adolescent/Young Adult Cancer Survivors: A Mixed Methods Study

**DOI:** 10.1002/pon.70272

**Published:** 2025-09-13

**Authors:** Pooja Rao, Katie A. Devine, Kristin Bingen, Allison M. Scott, Laura M. Koehly, Courtney L. Rumbaugh, Emily Wasserman, Heather J. Costigan, Smita Dandekar, Natthapol Songdej, George F. Blackall, Eugene J. Lengerich, Lauren J. Van Scoy

**Affiliations:** ^1^ Division of Pediatric Hematology/Oncology Department of Pediatrics Penn State Health Children's Hospital Hershey Pennsylvania USA; ^2^ Rutgers Cancer Institute New Brunswick New Jersey USA; ^3^ Division of Pediatric Psychology and Developmental Medicine Department of Pediatrics Medical College of Wisconsin Milwaukee Wisconsin USA; ^4^ Department of Communication University of Kentucky Lexington Kentucky USA; ^5^ Social and Behavioral Research Branch National Human Genome Research Institute National Institutes of Health Bethesda Maryland USA; ^6^ Penn State College of Medicine Hershey Pennsylvania USA; ^7^ Penn State Cancer Institute Hershey Pennsylvania USA

**Keywords:** adolescent/young adult cancer survivor, mixed methods, psycho‐oncology, psychological health

## Abstract

**Background:**

Adolescent/young adult cancer survivors (AYACS), describing people diagnosed with cancer between 15 and 39 years old across the cancer continuum, suffer from poor psychological health. Poor psychological health is associated with difficulty achieving professional goals, financial stress, and poorer health.

**Aims:**

The objective of this study was to use a mixed methods approach to understand AYACS’ perspectives on how a personal cancer diagnosis impacts psychological health.

**Methods:**

In this convergent mixed methods study, data from 35 AYACS participants were collected consisting of Patient Reported Outcomes Measurement Information System Anxiety and Depression measures, a sociodemographic survey, and semi‐structured interviews.

**Results:**

Mixed methods integration revealed the following for AYACS across the cancer continuum: (1) A need for proactive and longitudinal addressal of psychological health; (2) Promotion of social connectedness as a means of coping with illness; (3) A need for innovative and age‐appropriate coping strategies; and (4) Promotion of resilience to help improve psychological health.

**Conclusions:**

This study provides direction for intervention development to improve psychological health.

## 1 Background

Adolescent/young adult cancer survivors (AYACS) are people diagnosed with cancer between 15 and 39 years of age, including those receiving and those who completed treatment. Treatment advances have resulted in 5‐year survival rates of 80%–90% [1], yet AYACS’ psychological health (encompassing emotional and mental health) suffers long into survivorship [2]. AYACS’ struggle with psychological health compared to peers is related to disrupted psychological development from cancer and its treatment [3, 4]. Poor psychological health is associated with difficulty achieving professional goals [5], financial stress [6], and poorer health [7].

While programming supporting psychological health may be offered to AYACS at academic centers (e.g., social work, psychology referrals, support groups), limited service uptake indicates newer strategies addressing psychological health are needed. Moreover, as most AYACS receive care in community settings [8], cost‐effective, accessible approaches are imperative. The study's objective was to use a mixed methods approach (i.e., collecting and integrating quantitative and qualitative data) to understand AYACS’ perspectives on how a personal cancer diagnosis impacts psychological health, providing a direction to develop psychological health interventions.

## 2 Methods

### 2.1 Participants

This mixed methods analysis was part of a larger study assessing AYACS’ psychosocial, professional and financial challenges. Participants were AYACS 15–25 years old at time of cancer diagnosis and within six years of cancer diagnosis. This AYACS subset was chosen to focus on those undergoing similar life challenges (i.e., higher education preparation/completion, shaping career goals, planning financial independence) that could impact psychological health [2]. Participants were receiving or received oncologic care at Penn State Health, a tertiary care center. Additional inclusion criteria included English fluency and computer or smartphone access. Participants who were non‐English‐speaking were excluded due to available study team resources. Participants with relapsed cancer or with cognitive or physical inability to participate were excluded to ensure these characteristics would not confound exploration of factors potentially impacting psychological health.

### 2.2 Recruitment

Data collection occurred Winter 2021‐Summer 2022, with remote recruitment conducted from study start until early 2022 when COVID‐19 study precautions were lifted and in‐person recruitment was renewed. Participants were identified through review of the institutional cancer registry, divisional cancer database, electronic medical record (EMR), and clinic templates. Enrollment was stratified to include participants representing varied ages at cancer diagnosis (i.e., 15–21 years old or 22–25 years old) and treatment phases (i.e., receiving or completed cancer treatment). We expected data saturation at 20 interviews based on pilot work [9] but aimed to recruit 35–40 participants to ensure adequate representation by age and cancer treatment phase.

Participants were approached for consent and assent (if < 18 years old) remotely or in‐person. The study team reviewed the summary of explanation of research with the participant (and parent/guardian of minors). If they agreed to participate, the participant verbally consented. For minors, verbal assent was obtained in addition to verbal consent by the parent/guardian.

### 2.3 Quantitative Data Collection

Demographic data was collected via a questionnaire and EMR review. Quantitative data related to psychological health was collected using Anxiety (Emotional Distress‐Anxiety‐Short Form 8a) and Depression (Emotional Distress‐Depression‐Short Form 8b) measures from the Patient Reported Outcomes Measurement Information System (PROMIS) [10]. Each scale yields a standardized *T* score, with a reference population mean of 50 and standard deviation of 10, using their scoring service [11]. Higher scores indicate higher symptoms. PROMIS measures have been used to assess depression and anxiety among AYACS [12].

### 2.4 Qualitative Data Collection

Two experienced qualitative interviewers (CR, HC) utilized a semi‐structured interview guide (Supporting Information S1: Appendix 1) to conduct individual participant videoconference interviews. The interview guide assessed psychological health and psychosocial resource utilization following a personal cancer diagnosis. Questions were informed by literature review and authors' experiences speaking with AYACS about psychological health. Interviews lasted 30–45 min, were audio‐recorded, and transcribed verbatim by a professional transcription service. Data (i.e., interview recordings and transcripts) were stored and managed using REDCap.^1^

### 2.5 Quantitative Data Analysis

Descriptive statistics for demographic characteristics were summarized using mean, standard deviation, median, and ranges for continuous measures. Two‐sample independent *t*‐tests assuming equal group variances and significance level of 0.05 were used to compare differences in mean anxiety and depression *T* scores based on sex (female vs. male), cancer diagnosis age (15–21 years vs. 22–25 years) and treatment phase (completed vs. receiving treatment). Statistical programming and analyses were performed using SAS software Version 9.4.

### 2.6 Qualitative Data Analysis

Thematic analysis was used to analyze qualitative data using a phenomenological approach [13] to explore how a personal cancer diagnosis impacted AYACS’ experiences related to psychological health. MAXQDA software [14] managed data. Research team members with qualitative research experience reviewed approximately 10%–20% of the entire dataset to inductively create preliminary categories, codes, and subcodes describing the data. A preliminary codebook for “Psychological Challenges” and its related codes was constructed and used to code five transcripts, after which discrepant codes were adjudicated by group discussion to create a final codebook with codes, definitions, and exemplars. Two coders then coded the entire dataset using the constant comparison method [15], which involved coding transcripts in batches and meeting for calibration every 10 transcripts. To maintain coding rigor, inter‐rater reliability between coders was calculated and coding reconciled at each calibration timepoint and cumulatively to ensure the intraclass coefficient was greater than 0.7. The study team then reviewed coding to develop themes. Frequency count [n, coding frequency of each code/subcode (once per participant transcript)] were abstracted from MAXQDA.

Qualitative themes related to psychological health are presented in this paper. Quotes were edited minimally for clarity and brevity. See Supporting Information S1: Appendix 2 for COREQ reporting guidelines/codebook, and Supporting Information S1: Appendix 3 for additional quotes.

### 2.7 Mixed Methods Integration

A convergent mixed methods approach explored how psychological health was impacted by a personal cancer diagnosis. To integrate findings, we used merging, where qualitative data was analyzed separately from quantitative data. Both findings were then compared and contrasted by the research team in a joint display [16]. A median sample split was completed for Anxiety and Depression *T* scores to descriptively compare thematic findings by those who reported lower anxiety or depression symptoms (below median) versus higher anxiety or depression symptoms (above median). Thematic findings, coding content and participant coding frequency counts (*n*) were descriptively compared based on sex, diagnosis age, and treatment phase.

## 3 Results

### 3.1 Participant Demographics

Seventy‐six participants were approached and 41 enrolled (53.9%). Six withdrew before study data were collected. Withdrawal reasons included logistics (i.e., participants too busy to participate) and being lost to follow‐up prior to the study visit. Thus, data were collected from 35 participants (Table 1). Quotes were analyzed from 34 participants given one participant's interview was unintentionally not audio‐recorded due to technical difficulties; these 34 participants were included in mixed methods integration. About half of participants were male (51.4%). Most participants were White (82.8%) and not Hispanic/Latino (97.1%). Leukemias and lymphomas represented the greatest proportion of cancer diagnoses (74%). Most participants (63%) were post‐treatment survivors, enrolled in school (60%), and employed part‐time or full‐time (57%).

**TABLE 1:** Demographics.

	(*N* = 35)
Age at interview (years)	
Mean (SD)	22.5 (3.92)
Median	22.7
Range	16.4–29.1
Participant identified sex	
Female	17 (48.6%)
Male	18 (51.4%)
Race	
Asian/Pacific Islander	2 (5.7%)
Black/African American	2 (5.7%)
Hispanic/Latino	1 (2.9%)
Native American	1 (2.9%)
White	29 (82.8%)
Ethnicity	
Hispanic/Latino	1 (2.9%)
Not Hispanic/Latino	34 (97.1%)
Stratification	
15–21 years, receiving treatment	10 (28.6%)
22–25 years, receiving treatment	3 (8.6%)
15–21 years, completed treatment	12 (34.2%)
22–25 years, completed treatment	10 (28.6%)
Cancer diagnosis	
Acute lymphoblastic leukemia	9
Acute promyelocytic leukemia	1
Mixed phenotypic acute leukemia	1
Chronic myeloid leukemia	1
Hodgkin lymphoma^a^	13
Peripheral T cell lymphoma	1
Adrenocortical carcinoma	1
Breast cancer	2
Nasopharyngeal carcinoma	1
Ewing sarcoma	1
Soft tissue sarcoma (angiosarcoma)	1
Juvenile pilocytic astrocytoma	1
Malignant mixed germ cell tumor	1
Testicular cancer	1
Education	
Enrolled in high school	8 (22.9%)
Enrolled in college	10 (28.6%)
Enrolled in technical/professional school	1 (2.9%)
Enrolled in graduate school	2 (5.7%)
Not enrolled‐completed high school	4 (11.4%)
Not enrolled‐completed some college	1 (2.9%)
Not enrolled‐completed college	8 (22.9%)
Not enrolled‐ completed technical/professional school	1 (2.9%)
Employment	
Not currently employed	15 (42.9%)
Currently employed full‐time	12 (34.3%)
Currently employed part‐time	8 (22.9%)
Who do you live with? (check all that apply)	
Significant other	6 (17.1%)
Siblings	3 (8.6%)
Parents	13 (37.1%)
Parents and significant other	2 (5.7%)
Parents and siblings	7 (20.0%)
Other (lives alone, declined response, lives with other family members)	4 (11.4%)

^a^ One participant was diagnosed with Hodgkin lymphoma at age 24, and 2 years later was diagnosed with chronic myeloid leukemia.

### 3.2 Quantitative Results

The median anxiety *T* score was 56 [Interquartile range (IQR) 12.58], and the median depression *T* score was 47.3 (IQR 15.75). No statistically significant differences in anxiety or depression *T* scores were noted when stratified by sex, diagnosis age, or treatment phase (Table 2).

**TABLE 2:** *T*‐tests of PROMIS *T* scores.

	Mean (SD)	Mean (SD)	Difference (95% CL)	*p*‐value
	(95% CL) *n*	(95% CL) *n*		
	**Female**	**Male**		
PROMIS anxiety	56.3 (6.6)	52.3 (8.5)	4.0 (−1.3, 9.2)	0.1
	(52.9, 59.7)	(48.1, 56.5)		
	17	18		
PROMIS depression	49.7 (7.5)	45.2 (7.3)	4.4 (−0.7, 9.5)	0.09
	(45.8, 53.5)	(41.6, 48.9)		
	17	18		

	Age 15‐21	Age 22‐25		
PROMIS anxiety	54.7 (8.5)	53.4 (6.7)	1.4 (−4.3, 7.0)	0.6
	(51.0, 58.5)	(49.4, 57.4)		
	22	13		
PROMIS depression	48.0 (8.4)	46.4 (6.4)	1.5 (−3.9, 7.0)	0.6
	(44.2, 51.7)	(42.5, 50.3)		
	22	13		

	Not currently receiving cancer treatment	Currently receiving cancer treatment		
PROMIS anxiety	53.0 (7.5)	56.3 (8.2)	−3.3 (−8.8, 2.2)	0.2
	(49.7, 56.3)	(51.4, 61.3)		
	22	13		
PROMIS depression	45.5 (7.0)	50.5 (7.9)	−5.0 (−10.2, 0.2)	0.06
	(42.4, 48.6)	(45.7, 55.3)		
	22	13		

### 3.3 Qualitative Results

Five themes emerged:

#### 3.3.1 Theme 1: Participants Described Various Uncomfortable Emotions Throughout the Cancer Continuum That Were Difficult to Manage

Participants, irrespective of sex, diagnosis age or treatment phase, reported various uncomfortable emotions. Many reported sadness as expected after a cancer diagnosis. However, for some those initial emotions persisted:

So having the extreme lack of motivation to do anything, compiling with not seeing your work done, it's just piling up the more you let time pass, was kind of stressful to say the least. It's the fact that you know it's piling up and you can't do much about it.

(Participant 3, 15–21 years old and receiving treatment, Acute lymphoblastic leukemia, anxiety *T* score 43.2, depression *T* score 43.1)

Others described lingering stress even after completing therapy:

It was just really hard with, like, the depression and… rolling into, like, this off of treatment, into, like, the normal lifestyle, which I wasn't really adapting very well.

(Participant 34, 22–25 years old and completed treatment, Peripheral T cell lymphoma, anxiety *T* score 51, depression *T* score 37.1)

There were similar coding frequencies for negative emotions for those with higher and lower anxiety symptoms, and those with higher and lower depression symptoms. There was higher coding frequency for experienced negative emotions for younger (*n* = 19) compared to older participants (*n* = 11), and for those off treatment (*n* = 18) versus on treatment (*n* = 12). There were no coding content differences of negative emotions based on sex, diagnosis age, or treatment phase. Coding content differences by treatment phase were challenging to discern given difficulty timing occurrence of past and current emotions for participants off treatment.

#### 3.3.2 Theme 2: Participants Described Various Ways of Coping With Negative Emotions Associated With Their Personal Cancer Diagnosis And/Or Treatment

Younger and older participants relied on various methods to cope with cancer and its side effects. Distraction was a frequently cited coping strategy. Younger participants tended to describe how expressive therapy, such as music and art therapy, served as a welcome distraction:

… whenever, you know, I was inpatient in the hospital, I had a lot of trouble sleeping and relaxing… just because my body was so uncomfortable. You know, pain in my hips and in the joints… So they were able to just kind of take my mind away from that with their music therapy.

(Participant 11, 15–21 years old and completed treatment, Acute lymphoblastic leukemia, anxiety *T* score 60.8, depression *T* score 50.5)

Older participants (who had lower coding frequencies (*n* = 3) compared to younger participants (*n* = 14)) tended to describe personal hobbies as a distraction coping strategy:

… One of my therapy… we have motorcycles… recently… not long before I was diagnosed I bought another one and I was working on it. And… that helped calm me and relax me… riding motorcycles… It sounds crazy, but… you know, when you start right, you just black out. You don't, you don't think of anything. I mean, you're all there. You're not going to wreck, you know what I mean?

(Participant 27, 22–25 years old and completed treatment, Hodgkin lymphoma, anxiety *T* score 49.4, depression *T* score 52.5)

Coding content differences on coping by treatment phase were challenging to discern given difficulty timing occurrence of past and current coping strategies for participants off treatment.

Connecting with peers was a common coping strategy across sex, diagnosis age, and treatment phases. Participants described technology to connect with social contacts:

I mean, even after I got out, I went to… this restaurant and bar that we usually hang out with to see everybody after I got out. You know, I wanted to get out.

(Participant 27, 22–25 years old and completed treatment, Hodgkin lymphoma, anxiety *T* score 49.4, depression *T* score 52.5)

Females described coping through expressive therapies more commonly than males, who commonly described avoidance strategies—staying positive by not thinking about their cancer diagnosis—and hobbies like videogaming. No coding content differences were noted based on lower versus higher anxiety and depression symptoms.

#### 3.3.3 Theme 3: Professional Psychological Services Were Often Declined, Although Considered By Many

Younger and older participants described declining professional help from therapists, psychologists, or other sources during and after cancer treatment, even when services were offered. Reasons for declining included being ill‐equipped to receive help, and feeling such services were not needed. Yet, several who completed treatment described how in hindsight they would have reconsidered this help:

Therapist is something that had come up… with my doctor after my initial diagnosis… at that point I don’t know if it—where I was at the time, it wasn’t something that I thought I needed…as the years have gone by, maybe… I've considered… trying to re‐initiate that sort of conversation, as it might be useful.

(Participant 30, 22–25 years old and completed treatment, Testicular cancer, anxiety *T* score 46.4, depression *T* score 46)

Participants who pursued professional help described finding it helpful to have someone to talk to who was not closely related to them:

But it's really nice that I get to talk to someone… and other than, you know, squawking to my parents…it‐‐ it's nice that I get to talk to someone that, and talk through my feelings and, you know, just be supportive in that way… And [the psychologist is] a really nice and cool dude.

(Participant 31, 15–21 years old and receiving treatment, Acute lymphoblastic leukemia, anxiety *T* score 48.1, depression *T* score 37.1)

Another alluded to learning strategies like self‐efficacy:

…. I got a little bit of support for [depression with medication]. And that‐‐ that definitely did help as well. That was a big thing of being able to manage it, or at least, like, getting to a point where I could start to manage it myself.

(Participant 40, 15–21 years old and completed treatment, Hodgkin lymphoma, anxiety *T* score 54.6, depression *T* score 48.3)

No coding content differences related to using professional psychological services were observed from participants with higher or lower anxiety and depression symptoms, or across sex, diagnosis age, or treatment phase. An increased count frequency related to this theme was found for younger (*n* = 18) versus older participants (*n* = 9), and for those off treatment (*n* = 17) versus those on treatment (*n* = 10).

#### 3.3.4 Theme 4: Social Isolation and Loneliness Were Noted as Consequences of Physical Isolation Related to Treatment, Impacting Psychological Health

Being physically separated or isolated from others, whether due to distance, cancer treatment, side effects, or the COVID‐19 pandemic, led to social isolation and impacted psychological health. One participant compared their loneliness to isolation experienced during the COVID‐19 pandemic:

But I think… it was just lonely. And I think the world for the past two years, everyone's kind of felt that… regardless of whatever you're going through.

(Participant 36, 22–25 years old and completed treatment, Hodgkin lymphoma, anxiety *T* score 58.2, depression *T* score 46)

Another concurred with feelings of loneliness due to being physically isolated due to cancer treatment:

I mean, it's isolation. It's isolating, but like just in terms of mental health, it's really hard to not even be‐‐ a room that's now your own room. I think that I wasn't prepared necessarily for how like just kind of like on my own it would feel or like surreal, even.

(Participant 5, 22–25 years old and completed treatment, Acute lymphoblastic leukemia, anxiety *T* score 57.5, depression *T* score 50.8)

More participants who completed treatment contributed to this theme's content. Slightly more participants with higher anxiety and depression symptoms contributed to this theme. Coding content was similar across sex, diagnosis age, and treatment phase.

#### 3.3.5 Theme 5: Despite Experiencing Negative Emotions During Their Cancer Journey, Participants Also Described Resilience and Optimism

Despite experiencing negative emotions, participants described optimism for the future and appreciating their life with a new perspective:

… it sounds strange, but I am happier, in a way, because it feels like I actually have some sort of direction I'm working towards now. Like, it feels like I‐‐ I understand that my time on this Earth is limited. And now that I understand that, I feel like what I am doing does have a little bit more purpose, even though I don't know what that purpose is. So don't get me wrong, if I could go back in time and never get diagnosed, I absolutely would take that decision. But… there are a little, little positive bits sprinkled throughout my diagnosis that keeps me moving forward.

(Participant 22, 15–21 years old and receiving treatment, Acute lymphoblastic leukemia, anxiety *T* score 67.8, depression *T* score 59.5)

Another concurred with acceptance of the future's uncertainty:

… if one day I, you know, have a relapse or whatever may happen, then there's a time for that. But right now, I'm like very… I feel like I can see the world in full color.

(Participant 36, 22–25 years old and completed treatment, Hodgkin lymphoma, anxiety *T* score 58.2, depression *T* score 46)

More males contributed to this theme. A higher proportion of participants with higher anxiety symptoms (but not depression) compared to lower symptoms contributed to this theme. Coding content was similar across sex, diagnosis age and treatment phase.

### 3.4 Mixed Methods Integration and Joint Display

See joint display findings (Table 3). Qualitative and quantitative data were organized and interpretations and conclusions were discussed by the research team. See Discussion for joint display interpretations.

**TABLE 3:** Joint display.

Qualitative	Quantitative	Interpretation
**– Theme 1**: Participants described various uncomfortable emotions throughout the cancer continuum that were difficult to manage. **– Theme 2**: Participants described various ways of coping with negative emotions associated with cancer diagnosis and/or treatment. – Older participants (who had lower coding frequencies (*n* = 3) compared to younger participants (*n* = 14)) tended to describe personal hobbies as a distraction coping strategy – Females described distraction through music/art therapy services more commonly than males, whereas males more commonly described avoidance strategies. **– Theme 3:** Professional psychological services were often declined, although considered by many. – An increased count frequency related to this theme was found for younger (*n* = 18) versus older participants (*n* = 9), and for those off treatment (*n* = 17) versus those on treatment (*n* = 10).	– No significant differences in anxiety or depression *T* scores when stratified by sex, age at diagnosis, or treatment phase	– Proactively normalizing and addressing psychological health is necessary both during and after treatment. – Need for innovative and age‐appropriate coping strategies for AYACS
**Theme 4:** Social isolation and loneliness were noted as consequences of physical isolation related to treatment, impacting psychological health. – More participants who completed treatment contributed to this theme's content.	– A higher proportion of participants with higher anxiety and depression symptoms contributed to this theme	– Social connectedness as a means of distraction and coping with illness and treatment throughout the cancer continuum – Leveraging age‐appropriate technologies to connect with others
**Theme 5:** Despite experiencing negative emotions during their cancer journey, participants also described resilience and optimism.	– A higher proportion of participants with higher anxiety symptoms contributed to this theme	– Promotion of AYACS’ resilience to improve psychological health – Role of peer support to promote posttraumatic growth

## 4 Discussion

This mixed methods analysis adds to existing literature to understand AYACS’ perspectives on how a personal cancer diagnosis impacts psychological health. While our cohort endorsed average levels of depression and anxiety compared to the general population, qualitative data provides deeper examination of quantitative findings by exploring the complex emotions experienced after a personal cancer diagnosis throughout the cancer continuum, with descriptions of various negative emotions irrespective of sex, age, or treatment phase. Interestingly, while literature supports increased incidence and prevalence of anxiety and depression among females compared to males [17], we did not find a statistically significant difference in anxiety and depression *T* scores by sex. Notably, there were no qualitative differences related to experienced emotions, seeking of professional help, social isolation, resilience or optimism revealed across sex, diagnosis age and treatment phase, indicating feelings of psychological stress are universal for these AYACS.

### 4.1 Clinical and Research Implications

There is a paucity of survivorship/supportive care studies included in NCI's Evidence‐Based Cancer Control Programs [18]. Our mixed methods integration (Table 3) provides direction to develop recommendations to improve AYACS’ psychological health in clinical settings, and future research.

#### 4.1.1 Proactive and Longitudinal Addressing of Psychological Health is Critical for All AYACS

We recommend screening AYACS’ psychological health throughout the cancer continuum. Many post‐treatment survivors recalled psychological stressors. Previous research supports the lasting effects of cancer‐related stress long into survivorship [19]. Post‐treatment survivors are seen less frequently by their oncology team compared to those receiving treatment, thus opportunities to discuss professional psychological help are reduced. Moreover, as many AYACS are integrating into professional endeavors after treatment completion [20], they may need additional psychological supports to contend with these challenges and at these junctures in their cancer care.

Accessing psychological support continues to be an unmet need for many AYACS, especially minoritized populations [21]. Yet, our study highlighted how many AYACS were offered, yet declined professional help. Those amenable to seeking professional help (often after completing treatment) found it helpful for coping with illness. A great deal of stigma surrounds psychological health, varying by culture and context [22]. This stigma exists despite improving trends in psychological health literacy (i.e., knowledge of psychological health, and skills to seek support if needed) [23] among young people [24]. Future research can focus on understanding barriers and facilitators of seeking professional help, conveying practical benefits, and normalizing psychological support as part of AYACS care. Given many participants found such support helpful, even promoting self‐efficacy to cope with illness, finding ways for such help to be more approachable (even if available) is needed.

#### 4.1.2 Social Connectedness Should Be Promoted as a Means of Coping With Illness Among AYACS Throughout the Cancer Continuum

Social isolation and loneliness were consequences of physical isolation related to treatment and its side effects, impacting psychological health, with a slightly higher proportion of participants with higher anxiety and depression symptoms contributing to this theme. Our team has identified the importance of social connection to cope with illness, and how important relationships may change due to a personal cancer diagnosis, corroborated by literature [25, 26]. Social connection has emerged as a renewed focus for health in the general population, and in cancer organizations [27, 28]. Additionally there is evidence on leveraging age‐appropriate technology like video gaming within the AYACS community as a means to cope and connect with others [29]. Having opportunities to connect with others through virtual methods could be helpful to improve psychological health and explore in clinical programming, while addressing factors like immunosuppression‐related concerns and travel. We found virtual social connection can promote coping [30] and adoption of healthy behaviors like exercise [9]. Moreover, our findings highlight social buffering theory whereby social connections can reduce responses like stress [31].

#### 4.1.3 There is a Need for Innovative and Age‐Appropriate Coping Strategies for AYACS

Our study demonstrated how participants rely on various coping methods. Pediatric oncology programming largely focuses on Child Life services and expressive therapies, which younger participants often accessed and benefited from. There is a need for tailored, cost‐effective clinical programming for AYACS to address psychological health, especially older AYACS who may not have access to, nor be the intended audience of this type of pediatric programming. Programs like events and retreats focused on AYACS’ hobbies and interests may benefit older AYACS and potentially have added benefit of social connection, which was lacking for many participants. Some centers have AYA‐dedicated psychosocial programming, and are available virtually which may be appealing to some AYACS [30]. As females found distraction through expressive therapies more so than males, who described focusing on positives in life and not thinking about cancer, and hobbies like videogaming, these differences may help tailor approaches for AYACS in cancer care.

#### 4.1.4 Promoting Resilience Could Help Improve Psychological Health Among AYACS

Despite stressors throughout the cancer continuum, participants' insights into impacts of a personal cancer diagnosis demonstrate their optimism and resilience in the face of adversity. Interestingly, a higher proportion of participants with higher (compared to lower) anxiety symptoms contributed to Theme 5, suggesting AYACS may experience higher anxiety symptoms, yet still hold optimism for their future. Theme 5's findings are closely linked to the posttraumatic growth concept, whereby people can experience positive outcomes as a result of trauma, including emotional growth, and a greater appreciation for life. Promising work on building AYACS’ resilience [32] could also have discernable impact on improving psychological health. Additionally, research leveraging AYACS peer support to foster posttraumatic growth [33] is promising. Leveraging AYACS’ peer support to promote health is an active area of investigation within our group [26].

### 4.2 Strengths and Limitations

A strength of this study is a mixed methods design, which allowed us to capture qualitative data to further understand constructs of psychological health and how these data map onto our quantitative measures. Additional strengths included collecting data from AYACS representing a broad range of ages, treatment phases, and diagnoses. We had robust male representation, which is significant as males are underrepresented in AYACS psychosocial studies [34]. We had a high proportion of patients with leukemias and lymphomas, representative of the significant proportion of these malignancies among AYACS [35]. About two‐thirds of participants were post‐treatment survivors, which let us capture a rich perspective from those who could reflect on their experience over the cancer continuum.

Limitations included being a single center study with a majority of White/Not Hispanic participants, so our results may not be generalizable to areas that are demographically different by race, ethnicity, or other sociodemographic variables. Our participants' median anxiety and depression *T* scores were lower than what may be considered moderate or severe anxiety and depression, which could influence data interpretations in different settings. Our study focused on younger AYACS so results may not generalize to older AYACS 26–39 years old. Additionally, as most participants were diagnosed with hematologic malignancies, our study may be insufficient to identify psychological health challenges for AYACS with other cancer types. Our qualitative results from post‐treatment survivors may have conflated both current and past psychological challenges. Additionally, frequency counts in theming analysis were not intended to be precise due to nature of qualitative analyses, but rather to help uncover general patterns and lend support to our themes. Our institution benefits from philanthropic funding for those diagnosed with cancer prior to their 22nd birthday with Child Life and expressive therapies, Psychology, and Social Work services, which could have impacted psychological challenges of younger AYACS. Lastly, this was a small quantitative sample which could affect generalizability.

## 5 Conclusion

AYACS across sex, age, and treatment phase experience substantial psychological challenges. This study provides direction to develop psychological interventions with a focus on longitudinal psychological care for all AYACS, strategies leveraging technologies to promote psychological health, destigmatization and normalization of psychological health, and fostering social connectedness and resilience to improve psychological health.

## Author Contributions

P.R., L.J.V.S., K.B., E.J.L. and G.F.B. contributed to study conception and study design, as well as funding acquisition. *Material preparation, data collection and analysis were performed by P.R., L.J.V.S., C.L.R., H.J.C. and E.W.* The first draft of this manuscript was written by P.R. All authors read and approved the final manuscript. The data generated during and/or analyzed during the current study are not publicly available, nor are they available on request due to due to preserving participant anonymity.

## Ethics Statement

This study was performed in line with the principles of the Declaration of Helsinki. Approval was granted by the Institutional Review Board at Pennsylvania State University (STUDY00015658).

## Consent

This study was considered exempt research by the Institutional Review Board at Pennsylvania State University. The research team member reviewed the summary explanation of research (SER) with the participant. After review, the participant (or participant's parent/guardian) provided verbal consent prior to any study activities occurring. Before interviews were conducted, participants were recorded verifying that they reviewed the SER and agreed being recorded.

## Conflicts of Interest

The authors declare no conflicts of interest.

## Supporting information


Supporting Information S1

## Endnotes

^1^
Supported by National Center for Advancing Translational Sciences, National Institutes of Health, through Grant UL1 TR002014 & Grant UL1 TR00045.
