# Supplementary material for: The Impact of a Personal Cancer Diagnosis on the Psychological Health of Adolescent/Young Adult Cancer Survivors: A Mixed Methods Study
Source: Psychooncology. 2025 Sep 13;34(9):e70272. doi: 10.1002/pon.70272 (PMC12433271; doi:10.1002/pon.70272)
Supplement: Supplementary file 1 — Supporting Information S1 [file PON-34-e70272-s001.docx]

**Appendix 1: Interview Guide**

**Aim 1: Qualitatively explore the psychosocial challenges and resource utilization of AYA cancer**

**survivors as they relate to anxiety, depression, financial toxicity**

- OPENING: We know that in addition to the medical issues that go with being diagnosed and

treated for cancer, many patients your age experience a great deal of other challenges both during

cancer treatment and after. How has your experience with cancer treatment affected you?

- PROMPTS IF NEEDED:

• Have you experienced any psychological challenges? If yes, tell me more about that.

• Have you ever experienced any challenges related to mental health? If so, can you

tell us about that?

• Have you ever received any formal mental health diagnoses? Tell me more about

that.

- - Many patients your age say that their relationships with family, friends or others in their lives changed after they were diagnosed with cancer. Did having cancer change your relationships with family, friends or others in your life? How or how not?
  - Did any of your healthcare providers ever ask you how your relationships with family, friends or others in your life were going during cancer treatment, or after cancer treatment was finished? If so, do you remember who asked about that?
  - Do you feel that connecting with other people your age who have also gone through cancer treatment would be helpful or an additional support during cancer treatment or after cancer treatment? Why or why not?
- Many patients use different services such as a social worker, psychologist, or chaplain during

cancer treatment and after. Have you ever used these services? What was your experience using

these services?

- Are there any services that you wish you had available to you that were not offered to you?

Explain.

- We are interested in learning more about how cancer treatment affects young patients and

families with regards to finances. Is this something that affects you? How so?

- PROMPTS IF NEEDED:

• How, if at all, has your cancer impacted yours or your family’s finances?

• Have financial issues been something you and your family have talked about?

• What services, if any, have you received during cancer therapy or after to help

reduce financial burdens?

**Aim 2: Qualitatively explore how AYA cancer survivors’ experiences with cancer treatment**

**affect educational and occupational aspirations**

- OPENING: We know that many patients your age say that school can be challenging to do during

and after cancer treatment. How, if at all, has your cancer treatment affected your educational

goals?

- PROMPTS IF NEEDED:

• Tell me about any goals for school you had before the diagnosis of cancer?

• Did these goals for school change while you were being treated for cancer, or after?

If yes, how so?

• Did going through cancer treatment change what you wanted to do as far as

educational goals?

• How do you feel about going back to school?

• Do you feel supported in your return back to school after cancer treatment? If yes,

how so?

• For those who have completed treatment:

- After you finished cancer treatment, did you go to school? How soon

after? How was the process to integrate back to school? Is there

anything anyone could have done to make the process easier?

- OPENING: We know that many patients your age say that having a job can be challenging to do

during and after cancer treatment. How, if at all, has your cancer treatment affected your

occupational goals?

- PROMPTS IF NEEDED:

• Tell me about any goals for work you had before the diagnosis of cancer?

• Did these goals for work change while you were being treated for cancer, or after? If

yes, how so?

• Did going through cancer treatment change what you wanted to do as far as your

career?

• How do you feel about going back to work?

• Do you feel supported in your return back to work after cancer treatment? If yes,

how so?

• For those who have completed treatment:

- After you finished cancer treatment, did you go to work? How soon

after? How was the process to integrate back to work? Is there

anything anyone could have done to make the process easier?

**Appendix 2: COREQ Reporting Guidelines and Codebook**

**Domain 1: Research Team and Reflexivity**

*Personal characteristics:* Staff 1 is a research technologist with a B.S. in Biology and Psychology with a career interest in research dealing with behavioral psychology. Under the guidance of Staff 2, she conducted interviews for this study, with an understanding of the importance of research objectivity. Like Staff 2 she has prior experience conducting interviews of AYACS. Together with Staff 2 she coded and participated in theming of this study.

Staff 2 has multiple years of experience in qualitative and mixed-methods research and project management. Given her ample experience in conducting interviews and focus groups, she realizes the importance of objectivity during design and analysis as a result of possible bias with connections to the topic. She has prior experience working with the adolescent/young adult cancer survivor (AYACS) patient population and understands the sensitivity of the topics discussed in these interviews. She led the coding and theming of this study together with Staff 1.

The lead qualitative researcher (Staff 3) is an established physician scientist whose research interest focus on end of life and communication issues. Her qualitative work generally follows a pragmatic approach using descriptive methods to understand phenomena, and she applies a humanistic perspective to data analysis. She oversaw data theming and analysis.

Staff 4, a clinician-scientist, is the principal investigator of this project. Her research interests, largely informed by the clinical care she provides to AYACS, focuses on better understanding of the psychosocial issues of AYACS to devise effective interventions to improve long-term goal achievement and health-related behaviors of this patient population. Keenly aware of her potential for bias, she purposely did not participate in interview conduct for this study, nor the coding. She participated in theming for this study together with Staff 1 and Staff 2 who were closest to the data. Staff 1, 2, 3 and 4 all concurred on the final themes for this manuscript.

1. *Relationship with participants:* As Staff 4 either provides clinical care for or knows many of the AYACS in this study, she proactively did not conduct interviews or code the qualitative data, in order to maintain data objectivity. For this reason, Staff 1 and Staff 2 conducted all of the interviews and coding. Staff 1 and Staff 2 have prior experience performing interviews with this patient population.

**Domain 2: Study Design**

1. *Theoretical framework:* Thematic analysis was used to analyze the data. We used a phenomenologic approach that explored the phenomenon of psychological health and how a personal cancer diagnosis impacted AYACS’ experiences related to emotional and mental health. This approach avoids using preconceived categories and is appropriate to use when literature on a topic is limited.
2. *Participant selection:* Study participants were AYACS who were ages 15-25 years old at the time of cancer diagnosis and still within 6 years of their initial cancer diagnosis. Data collection occurred from the Winter of 2021 to the Summer of 2022. Recruitment was conducted remotely until early 2022 when COVID-19 precautions were lifted. This younger AYACS subset was chosen to study those undergoing similar developmental and interpersonal challenges to each other (e.g., developing autonomy and personal values/identity, cultivating peer relationships, preparing to enter the workforce). Participants were receiving or had received oncologic care at a tertiary care center that serves both urban and rural counties. Participants were recruited through review of the institutional cancer registry, the divisional cancer database, the electronic medical record, and clinic templates of providers. Additional inclusion criteria for participants (and consenting parents of participants < 18 years old) included being fluent in written and spoken English and having access to a computer or smartphone. Participants who were non-English speaking were excluded due to available study team resources. Participants with relapsed cancer or with cognitive or physical inability to participate were excluded to ensure these characteristics would not confound exploration of factors impacting psychological health.
3. *Setting:* After in-person recruitment, the study team and participant set up a mutual time over the videoconferencing platform Zoom to perform the study assessment. All data collection was conducted over Zoom.
4. *Data collection:* Two trained qualitative interviewers (Staff 1 and Staff 2) utilized a semi-structured interview guide to conduct one-on-one interviews with participants via videoconference. Interviews lasted approximately 30-45 minutes, were audio-recorded, and transcribed verbatim by a professional transcription service. The interview guide broadly explored several topics. The qualitative data related to psychological health is presented in this paper.

**Domain 3: Analysis and Findings**

1. *Data analysis:* Thematic analysis was used to analyze qualitative data using a phenomenological approach to explore how a personal cancer diagnosis impacted AYACS’ experiences relates to emotional and mental health. A phenomenological approach is a qualitative research methodology that seeks to understand a phenomenon (i.e. personal cancer diagnosis) by exploring it from the perspective of those who experienced it. This approach avoids using preconceived categories and is appropriate to use when literature on a topic is limited. The audio recordings were transcribed by 3 Play Media, a protected health information-approved transcription service. MAXQDA software was used to organize and manage analyses. Study team members with qualitative research experience or training (Staff 1, Staff 2) reviewed approximately 10-20% of the entire dataset to inductively create preliminary categories, codes, and sub-codes that described the data. The preliminary codebook was used to code five transcripts, after which the study team adjusted categories and codes to create the final codebook. Once established, two coders (Staff 1, Staff 2) used the final codebook to code the entire dataset using the constant comparison method which involved coding transcripts in batches and meeting for calibration every 10 transcripts. To maintain coding rigor, inter-rater reliability between the two coders was calculated and coding reconciled at each calibration timepoint and cumulatively to ensure intraclass coefficient was greater than 0.7. After coding, Staff 1 and Staff 2 developed initial themes, which Staff 4 further detailed. The full research team reviewed coding patterns together to agree on final themes. While the same analytic process was applied to the entire dataset, only themes related to the research question of psychological health (corresponding to code ‘Psychological Challenges’) are presented in this manuscript. Quotes were edited minimally for clarity and brevity.
2. *Reporting:* The manuscript discusses the themes that arose from coding and theming. Supporting participant quotes are provided. Quotes were minimally edited for clarity when appropriate.

**Psychological Health Codebook**

| Psychological Challenges | Subcode | Definition | Example quote |
| --- | --- | --- | --- |
|  | 1Ai. Formal Diagnosis | Mentions of a formal diagnosis before, during or after treatment | Uh, no. No, I'm not. I don't think-- I don't think I'm officially diagnosed with-- I'm not officially diagnosed with depression, I know that. I don't know about anxiety.  INTERVIEWER: OK.  SUBJECT: But they do have medicine for it, which is as needed. So it's not like I take it every single day. |
|  | 1Aii. General comments on mental health impacts | Mentions of mental health symptoms and impacts from diagnosis, treatment, and post-care | It was something that, um, my care team would ask me about, and kind of check-in on. Um. But, I mean, I think they did as much as they could and-- but there are times where it-it feels like you can't really control everything going into your health, and so uh, yeah. That-- that's challenging, and I-- I wish I had a solution for it, but at times things are just tough. |
|  | 1Aiii. Professional Help | Mentions of seeking/utilizing professional help | Uh, in the beginning, uh, I talked about the-- or in the question earlier, I talked about the psychologist. I didn't talk to him very much at all in the beginning because I didn't need to. But once I got to those rarer visits, I started talking to him more and more about how to control my anxiety and how to deal with problems that I was facing.  And whenever my mind would run away, he would, like, give you little strategies of how to cope with it and how to deal with things. And every once in a while-- I'm not proud to say it-- but there's like medicine, you know, prescribed obviously, that you take as needed for anxiety, which sometimes I've-- I've used. |
|  | 1Aiv. Mental Health Symptoms | Mentions of fears, anxiety, isolation, depression, etc. related to their diagnosis, treatment, after, | Um. It was kind of always from the beginning, it was like the-- I-- I realized that, you know, some of the anxieties and fears related to mortality would probably not be something that I just easily escaped or let go of when I finished treatment.  It's definitely a bit of a roller coaster, I would say. Like, I've never gotten, like, too bad. But there's definitely days where I'm just, like, oh, like, I'm-- I'm so scared. Like, I really don't want to go to treatment. Like, everything can just be kind of too much at times, I would say. |
|  | 1Av. Ways to distract from diagnosis/treatment | Mentions of ways to distract from thinking about cancer diagnosis, treatment, etc. | Yeah. I definitely try to distract myself. You know, like, find your own fun things that I can do for me. Like, recently I've been re-watching Hannah Montana. I don't know if you're familiar with the show.  But I would say-- what I-- I try to still keep as busy as I physically can. And when I can't, because, like, obviously-- chemo weeks most of the time, like, I'm not getting out of bed, you know. So uh, I would say those days. Yeah. I, I talked about FaceTiming. I talked about watching TV. I love TV. |

**Appendix 3: Themes and Supporting Participant Quotes**

| **Themes** | **Supporting Quotations** |
| --- | --- |
| **Theme 1: Participants described various uncomfortable emotions throughout the cancer continuum that were difficult to manage.** | |
| **Subtheme 1a:** Participants reported a variety of emotions related to the news of their cancer diagnosis. | “And it's [experience with cancer treatment] made me a little bit more anxious. And I've always been a little bit anxious. I've always been… a little bit more anxious.” (Participant 12, 15-21 years old and receiving treatment, Hodgkin lymphoma, anxiety T score 60.3, depression T score 56.3)  “…. I was… lost whenever I figured [my cancer diagnosis] out. Like I was confused. I thought it was a joke. And then I-- someone [with] a paper that said it. And I was like, oh. OK. So I wasn't fazed too much until later on and the stuff… After I found out it was real, ah, I was upset a lot.” (Participant 15, 15-21 years old and completed treatment, Hodgkin lymphoma, anxiety T score 48.3, depression T score 37.1)  “… emotionally it was just a shock, because it's not something you expect to hear after you have this lump on your neck for years. And it's just like, oh, yeah, that's cancer. It's just-- I don't know… I think it was harder for other people to hear that I-- I have cancer.” (Participant 17, 15-21 years old and receiving treatment, Nasopharyngeal carcinoma, anxiety T score 57.4, depression T score 55.5)  “I would say after being diagnosed, it got a lot worse. Because I don't really-- I never felt that way before. I was just kind of carefree, but now… I am worried about if what I am doing has purpose. I have that conversation with myself a lot. And a lot of times, the answer I find is like, no, what I am doing doesn't really have a purpose, or I'm not living a fulfilling life, or whatever.” (Participant 22, 15-21 years old and receiving treatment, Acute lymphoblastic leukemia, anxiety T score 67.8, depression T score 59.5)  “In the beginning, I was pretty-- I got depressed, I guess you can say. I was in a pretty dark place…It was more the why me kind of thing. And then I was looking at home, looking at the same four walls. And It just wasn't-- plus there's the initial shock. And I got diagnosed probably a month before my we-- or my wedding date. So that was-- yeah, that was quite the shock to us all.” (Participant 2, 22-25 years old and completed treatment, Hodgkin lymphoma, anxiety T score 48.3, depression T score 37.1)  “… it was definitely a big shock… you know, that, that whole week when I was diagnosed went so fast and I, I remember everything.” (Participant 27, 22-25 years old and completed treatment, Hodgkin lymphoma, anxiety T score 49.4, depression T score 52.5)  “… in the beginning, when I was, like, just in total complete shock that I'm like going through it, I was just-- I kind of like-- I totally, completely just trusted everything that [my cancer care team] were doing with me. But at the same time, I was just like, oh, I'm just… like, in just total shock of like the whole situation.” (Participant 34, 22-25 years old and completed treatment, Peripheral T cell lymphoma, anxiety T score 51, depression T score 37.1)  “So when I was, like, first diagnosed, I was kind of having, like, issues with anxiety anyway. Like, I think it might have stemmed from just, like, a college thing… like, the stress of college. And then cancer diagnosis on top of that was just, like, a lot. So I-- I started taking… medication for my anxiety. And it's helped so much. And, I think, if I wasn't on it, my mental state would be so much worse than it, like, is.” (Participant 35, 15-21 years old and receiving treatment, Hodgkin lymphoma, anxiety T score 43.9, depression T score 37.1)  “… it was kind of a shock, getting the diagnosis with having a brain tumor. That's kind of big news for a 16-year-old. So it was kind of difficult.” (Participant 7, 15-21 years old and completed treatment, Juvenile pilocytic astrocytoma, anxiety T score 65.6, depression T score 60.6)  “I don't know where to begin… challenges-- I was definitely, you know, I was blindsided and overwhelmed at first, for sure… the first, like, few months and even sporadically throughout… you know, it's just an overwhelming thing to deal with. It did bring a little anxiety, but I feel like it was, you know, a normal amount of anxiety for someone going through something like that.” (Participant 9, 15-21 years old and completed treatment, Acute lymphoblastic leukemia, anxiety T score 51, depression T score 37.1)  “I was, I went into instant tears…It was just the, the sadness… and the depression that came with it. And the stress.” (Participant 10, 22-25 years old and completed treatment, Breast cancer, anxiety T score 57.8, depression T score 50.4)  “I'm in a way better mindset now than in the beginning. It was a lot of depression, a lot of confusion, anger, hurtful. Like, you know, just feeling alone.” (Participant 23, 22-25 years old and receiving treatment, Hodgkin lymphoma, anxiety T score 47.3, depression T score 48) |
| **Subtheme 1b:** Participants reported a variety of emotions both during and after cancer treatment completion. | “And then I started to get, like, progressively more anxious. And, like, asked God why. I'm just, like, the longer, like, you get through your treatments, and like, the longer it takes, it should-- like, the more it, like, tolls on you. And, like, the longer you have to be, like, apart from your friends, and the longer you have-- it takes to [get] back to normal life, it just takes more and more.” (Participant 12, 15-21 years old and receiving treatment, Hodgkin lymphoma, anxiety T score 60.3, depression T score 56.3)  “So I guess the real psychological problems started a little bit later after everything kind of slowed down, and I got into like the later phases of my treatment where it was just going to-- going to… the doctor every month or whatever. Like I had less time to talk with people about it. So it felt like it built up a lot more like anxiety and stuff inside of me before I could talk to anyone about it.” (Participant 22, 15-21 years old and receiving treatment, Acute lymphoblastic leukemia, anxiety T score 67.8, depression T score 59.5)  “I think I'm just really overwhelmed. Um, like, the thought of doing more when I have, like, everything else trying to balance just seems like too much.” (Participant 29, 22-25 years old and receiving treatment, Breast cancer, anxiety T score 64.9, depression T score 57.1)  “I would say I was pretty depressed a lot throughout my treatment. But I-- I'd say I managed that pretty well.” (Participant 41, 15-21 years old and receiving treatment, Acute lymphoblastic leukemia, anxiety T score 61.4, depression T score 54.7)  “So having the extreme lack of motivation to do anything, compiling with not seeing your work done, it's just piling up the more you let time pass, was kind of stressful to say the least. It's the fact that you know it's piling up and you can't do much about it.” (Participant 3, 15-21 years old and receiving treatment, Acute lymphoblastic leukemia, anxiety T score 43.2, depression T score 43.1)  “[not feeling like myself during chemotherapy treatment] put me down. I think that's where I would get my most down moments and I needed the pick me uppers from my providers and family. Because I would get so happy because I'd finally not be throwing up anymore or not feeling weak. And I was able to run around, do… normal things I would do.” (Participant 13, 15-21 years old and completed treatment, Hodgkin lymphoma, anxiety T score 54.3, depression T score 48.3)  “Also, I definitely, now I feel like I have like a little bit of PTSD from what all happened. And like, also, sometimes going like back into the hospital, and like, remembering stuff, it's hard sometimes.” (Participant 21, 15-21 years old and completed treatment, Hodgkin lymphoma, anxiety T score 58.2, depression T score 44.5)  “But… there are times where my doctors could kind of tell that I was getting anxious or getting irritable or that kind of stuff.” (Participant 19, 15-21 years old and completed treatment, Acute lymphoblastic leukemia, anxiety T score 59.7, depression T score 46.6)  “I wouldn't say it was a down time. I did get angry. I think that was one-- one of the chemo drugs, I was-- I had a pretty short fuse. My wife's standing in the other room, and she could probably agree. I bit her head a couple times. And I probably shouldn't have. Yeah, I'm getting the look.” (Participant 2, 22-25 years old and completed treatment, Hodgkin lymphoma, anxiety T score 48.3, depression T score 37.1)  “… my family just wanted me to focus on my health. But at the same time… not, not be well to work had me thinking a lot about the cancer, and that drove me into, like, depression really fast.” (Participant 34, 22-25 years old and completed treatment, Peripheral T cell lymphoma, anxiety T score 51, depression T score 37.1)  “Like, I felt, like, hopeless. I felt hopeless.” (Participant 34, 22-25 years old and completed treatment, Peripheral T cell lymphoma, anxiety T score 51, depression T score 37.1)  “For the first few months, I didn't really adjust that much at all. But I mean, with how sick I was, I was basically just like either in the treatment center asleep all the time… so for a while, I-- I don't know that I really did adjust. I was just kind of stressed out and depressed a lot of the time.” (Participant 40, 15-21 years old and completed treatment, Hodgkin lymphoma, anxiety T score 54.6, depression T score 48.3)  “And then it's like I'm 20 years old, and then I-- I'm coming into this-- like, pediatric doctors, and I'm seeing these little babies. And I'm like-- it's-- that's really depressing, too, because they don't know what's going on. I'm old enough to understand. They're-- they're not old enough to understand it. I still get upset seeing those kids like that.” (Participant 8, 15-21 years old and completed treatment, Soft tissue sarcoma (angiosarcoma), anxiety T score 44, depression T score 53.9)  “… I think just, like, you know, the thing that I go back to when I think about it is just I really hated that my life was at a standstill… and I think-- like, I think at that point in my treatment it was the best thing for me. Because, you know, I didn't-- I was-- at the same time, I didn't want to risk getting sick or anything and, you know, prolonging anything or giving myself any more issues than I needed to have… so it made sense to me, but it still, like, kind of pissed me off a little bit.” (Participant 9, 15-21 years old and completed treatment, Acute lymphoblastic leukemia, anxiety T score 51, depression T score 37.1)  “The doctors told me that I had a very beatable type of cancer. But there's always that thought in the back of my head that what if I can't beat it.” (Participant 39, 15-21 years old and completed treatment, Malignant mixed germ cell tumor (retroperitoneum), anxiety T score 43.8, depression T score 37.1)  “It was just really hard with, like, the depression and… rolling into, like, this off of treatment, into, like, the normal lifestyle, which I wasn't really adapting very well.” (Participant 34, 22-25 years old and completed treatment, Peripheral T cell lymphoma, anxiety T score 51, depression T score 37.1) |
| **Theme 2: Participants described various ways of coping with negative emotions associated with their personal cancer diagnosis and/or treatment.** | |
| **Subtheme 2a:** Younger participants often sought distractions by engaging with expressive therapies. | “Most of the time, I would just either do the music therapy in the room or home work.” (Participant 13, 15-21 years old and completed treatment, Hodgkin lymphoma, anxiety T score 54.3, depression T score 48.3)  “… I'm not sure of her name, but she would come into my room and, like, play the guitar or, like, she built little clay snowmen with me, and that just-- I was in a lot of pain when I was in the hospital, so that, like, got my mind off of it, and just-- for that short period of time, it made a lot better.” (Participant 17, 15-21 years old and receiving treatment, Nasopharyngeal carcinoma, anxiety T score 57.4, depression T score 55.5)  “Well, [music therapy and other support services] was one of the-- it was just like kind of relaxing and calming. It got like my mind off of what was like going on around me with all like the machines and everything. It kind of just like let me focus on something. And just like hearing that, it soothed me.” (Participant 21, 15-21 years old and completed treatment, Hodgkin lymphoma, anxiety T score 58.2, depression T score 44.5)  “… I feel like I have so much time on my hands that, like, [school] was one of the things that I could just focus on. 'Cause I didn't have anything else to do, like, I might as well just do school.” (Participant 35, 15-21 years old and receiving treatment, Hodgkin lymphoma, anxiety T score 43.9, depression T score 37.1)  “And so usually, I would be sitting in the bed at the hospital in my little room with a laptop that I got from Make-A-Wish, and like, a mouse and a headset. And I would play video games just to, I guess, pass the time.” (Participant 37, 15-21 years old and completed treatment, Ewing sarcoma, anxiety T score 61.5, depression T score 54.5)  “… but it was just kind of finding the things that I could… spend my time with, like studying and things, or reading, or… just finding things to, like, engage me mentally so I wasn't just sitting there. You know?” (Participant 40, 15-21 years old and completed treatment, Hodgkin lymphoma, anxiety T score 54.6, depression T score 48.3)  “… whenever, you know, I was inpatient in the hospital, I had a lot of trouble sleeping and relaxing… just because my body was so uncomfortable. You know, pain in my hips and in the joints... So they were able to just kind of take my mind away from that with their music therapy.” (Participant 11, 15-21 years old and completed treatment, Acute lymphoblastic leukemia, anxiety T score 60.8, depression T score 50.5)  “Yeah, I definitely try to distract myself. You know, like, find own fun things that I can do for me. Like recently, I've been-- I've been re-watching Hannah Montana.” (Participant 24, 15-21 years old and receiving treatment, Hodgkin lymphoma, anxiety T score 62.7, depression T score 58)  “Music definitely always makes me feel better. So that's one of-- one of the coping mechanisms I would say I… go to” (Participant 24, 15-21 years old and receiving treatment, Hodgkin lymphoma, anxiety T score 62.7, depression T score 58)  “And it wasn't that bad because of, I'd say, mostly my friends, and just me getting my mind off of it by playing video games.” (Participant 37, 15-21 years old and completed treatment, Ewing sarcoma, anxiety T score 61.5, depression T score 54.5) |
| **Subtheme 2b:** Some participants reported hobbies and activities as a means of coping with their diagnosis and/or treatment. | “And I just kind of wanted to, I guess, walk away or block out everything that was going on around me. Yeah, I have-- I've done that a couple of times. Yes.” (Participant 22, 15-21 years old and receiving treatment, Acute lymphoblastic leukemia, anxiety T score 67.8, depression T score 59.5)  “Mentally, mentally, 100%. I could focus on-- like if a car I was working on, or something like that, took my mind off of cancer for a little bit, it just-- and the way our-- we've had some people, we've been in business 50 years. And there's been-- like, people have seen my dad [grow] up. They've seen me grow up. And then going in and just even talking to some customers, it, it really helped.” (Participant 2, 22-25 years old and completed treatment, Hodgkin lymphoma, anxiety T score 48.3, depression T score 37.1)  “… One of my therapy… we have motorcycles… recently… not long before I was diagnosed I bought another one and I was working on it. And… that helped calm me and relax me… riding motorcycles… It sounds crazy, but… you know, when you start right, you just black out. You don't, you don't think of anything. I mean, you're all there. You're not going to wreck, you know what I mean?” (Participant 27, 22-25 years old and completed treatment, Hodgkin lymphoma, anxiety T score 49.4, depression T score 52.5)  “I also love collecting action figures for Star Wars and stuff… but I'm more of a LEGO person now. So, you know, building is a big stress reliever. But I-- I know that during treatment and stuff, I've, you know, I have a few sets that I still haven't built in… just because A, I have neuropathy from my one… chemo, so I know that's a big thing, especially now, in like, my fingertips... It's-- it's weird… you know. I've-- they're-- they're sensitive, and you know, LEGOs are plastic and sometimes unforgiving. (CHUCKLES) So—so… you know, but I still-- I mean, my room's just filled with my LEGOs. Honestly, needing storage. But… yeah. I love looking at them and seeing what I've built… And I love fishing… We go trout fishing....” (Participant 31, 15-21 years old and receiving treatment, Acute lymphoblastic leukemia, anxiety T score 48.1, depression T score 37.1)  “… I started, like, going outside a lot more and doing things that were, like, just kind of, like, just to enjoy them, like just going on a walk for the sake of it, or, like, just kind of doing leisure activities, because a lot of the-- like before, I would basically pour, like, all of my time into working, like, pretty much all of the time that I was awake… and I-- I didn't really do a lot of things outside of it.” (Participant 40, 15-21 years old and completed treatment, Hodgkin lymphoma, anxiety T score 54.6 , depression T score 48.3) |
| **Subtheme 2c:** Some participants turned to social connections to distract themselves. | “Or, like, I'll FaceTime my friends just to, like, distract me, you know? New faces.” (Participant 24, 15-21 years old and receiving treatment, Hodgkin lymphoma, anxiety T score 62.7, depression T score 58)  “I mean, even after I got out, I went to… this restaurant and bar that we usually hang out with to see everybody after I got out. You know, I wanted to get out.” (Participant 27, 22-25 years old and completed treatment, Hodgkin lymphoma, anxiety T score 49.4, depression T score 52.5)  “[The social worker and I] wouldn't much talk about like my diagnosis and stuff. I guess he was trying to distract me. We'd talk about other stuff. Just whatever I was interested in, stuff like that.” (Participant 39, 15-21 years old and completed treatment, Malignant mixed germ cell tumor (retroperitoneum), anxiety T score 43.8, depression T score 37.1) |
| **Theme 3:** **Professional psychological services were often declined, although considered by many.** | |
| **Subtheme 3a:** Most participants thought about professional help, even if they did not pursue it. | “Uh, no. I-- I did not [seek out formal mental health while receiving treatment]…. And I've thought about that at times… I will say I, like, thought it would be interesting.” (Participant 1, 22-25 years old and completed treatment, Acute lymphoblastic leukemia, anxiety T score 64.6, depression T score 43.1)  “[when asked if seeking professional help would be something they would be interested in] I think about it sometimes… Because it's a lot to put on, like, my support system as it is, with just the diagnosis, let alone just letting them know how I feel.” (Participant 6, 15-21 years old and receiving treatment, Adrenocortical carcinoma, anxiety T score 61.3, depression T score 55.1)  “[Professional help] was offered, I just but-- I just didn't.” (Participant 13, 15-21 years old and completed treatment, Hodgkin lymphoma, anxiety T score 54.3, depression T score 48.3)  “They, they did offer counseling. But I never took it because I was… I had all the help that I needed throughout with those people.” (Participant 15, 15-21 years old and completed treatment, Hodgkin lymphoma, anxiety T score 48.3, depression T score 37.1)  “[when asked if interested in using psychological services] … sort of, but not really. I never really talked to somebody like that before, so I didn't know where to, like, begin with, I think.” (Participant 17, 15-21 years old and receiving treatment, Nasopharyngeal carcinoma, anxiety T score 57.4, depression T score 55.5)  “So… I feel like [psychological support] was-- like if I would have definitely asked for it, it would have been offered, but I just-- I feel, like I said, I didn't want to, you know, be a burden, I guess.” (Participant 11, 15-21 years old and completed treatment, Acute lymphoblastic leukemia, anxiety T score 60.8, depression T score 50.5)  “… and I have no problem talking about it now. Like people at work, they've asked me questions about it and all that kind of stuff, and I have no issues discussing it. It's just during the time it was just-- more so-- anger that was not targeted at anyone or anything. It was just more so I didn't understand.” (Participant 19, 15-21 years old and completed treatment, Acute lymphoblastic leukemia, anxiety T score 59.7, depression T score 46.6)  “… no professional help. I did have access to that, but I kind of, you know, stayed to myself praying all the time, you know?” (Participant 23, 22-25 years old and receiving treatment, Hodgkin lymphoma, anxiety T score 47.3, depression T score 48)  “Therapist is something that had come up… with my doctor after my initial diagnosis… at that point, I don't know if it*—*where I was at the time, it wasn't something that I thought I needed… as the years have gone by, maybe… I’ve considered… trying to re-initiate that sort of conversation, as it might be useful.” (Participant 30, 22-25 years old and completed treatment, Testicular cancer, anxiety T score 46.4, depression T score 46)  “And, like-- and if I needed to talk to anyone, I could just talk to [my support system]. I didn't feel like I really needed a professional for it.” (Participant 35, 15-21 years old and receiving treatment, Hodgkin lymphoma, anxiety T score 43.9, depression T score 37.1)  “I didn't take up the offer [to see a psychologist or therapist]. But now that I look back on it I wish I did…Just because I feel like as a teenager, it's very hard to get your emotions-- describe your emotions, and just understand them.” (Participant 37, 15-21 years old and completed treatment, Ewing sarcoma, anxiety T score 61.5, depression T score 54.5)  “Oh, well, I've never really naturally had any mental problems, so I didn't really need to [seek out psychological services] … But, no, I've never really needed a therapist.” (Participant 3, 15-21 years old and receiving treatment, Acute lymphoblastic leukemia, anxiety T score 43.2, depression T score 43.1)  “… I didn't seek any [psychological services] out.” (Participant 7, 15-21 years old and completed treatment, Juvenile pilocytic astrocytoma, anxiety T score 65.6, depression T score 60.6) |
| **Subtheme 3b:** Of the participants who received professional psychological help, they felt it to be beneficial. | “I didn't talk to [my psychologist] very much at all in the beginning because I didn't need to. But once I got to those rarer visits, I started talking to him more…And whenever my mind would run away, he would, like, give you little strategies of how to cope with it and how to deal with things. And every once in a while-- I'm not proud to say it-- but there's like medicine, you know, prescribed obviously, that you take as needed for anxiety, which sometimes I've-- I've used... it's like I need frequent checkups in order to keep myself in check, so [my psychologist] can keep reminding me of what I should be doing in order to control the stresses and anxieties I have.” (Participant 22, 15-21 years old and receiving treatment, Acute lymphoblastic leukemia, anxiety T score 67.8, depression T score 59.5)  “… I actually still probably, I'd say probably they're weekly, bi-weekly-- still meet with the psychologist that's down there at the hospital. So he's helped me quite a few times get through a lot of, like, rough patches and that kind of stuff. So, and, I mean, going through it and it teaching me a lot about, like, I can push a lot more than I think I can? Has got-- is the reason that I'm getting through school right now. I'm finishing up school. I'm making dean's list consistently. Like, this is the reason why.” (Participant 19, 15-21 years old and completed treatment, Acute lymphoblastic leukemia, anxiety T score 59.7, depression T score 46.6)  “… I love my therapist. I talk about her, like, all the time… she's been really helpful and she… we talk about my friend that, like, kind of, left and that's, like, a big thing that she's, like, really helped me with… it's just been nice to have someone that I know that I can, like, just talk about the crappy stuff.” (Participant 29, 22-25 years old and receiving treatment, Breast cancer, anxiety T score 64.9, depression T score 57.1)  “But it's really nice that I get to talk to someone… and other than, you know, squawking to my parents… it-- it's nice that I get to talk to someone that, and talk through my feelings and, you know, just be supportive in that way… And [the psychologist is] a really nice and cool dude.” (Participant 31, 15-21 years old and receiving treatment, Acute lymphoblastic leukemia, anxiety T score 48.1, depression T score 37.1)  “… I remember… still doing my maintenance chemotherapy and just feeling at my lowest, because… I was coming to an end with my chemotherapy maintenance treatments and I was feeling very depressed and very suicidal…[my doctors] did sign me up with the therapists that really helped me. Like, it just, it really, like… the therapist really helped me kind of, like, cope and transition into, like, kind of like adapted into, like, a normal lifestyle that I, like, have not had for a while.” (Participant 34, 22-25 years old and completed treatment, Peripheral T cell lymphoma, anxiety T score 51, depression T score 37.1)  “… I got a little bit of support for [depression with medication]. And that-- that definitely did help as well. That was a big thing of being able to manage it, or at least, like, getting to a point where I could start to manage it myself.” (Participant 40, 15-21 years old and completed treatment, Hodgkin lymphoma, anxiety T score 54.6, depression T score 48.3)  “And [the psychologist has] also been an amazing help with that…he was the one who, like, checked on me more than anything…It wasn't like I requested this with him. He, like, popped in, and I just told him what was going on and everything. But it really helped to talk to him then…Because it lets me get the feelings off my chest and everything, so-- I haven't talked to him recently.” (Participant 41, 15-21 years old and receiving treatment, Acute lymphoblastic leukemia, anxiety T score 61.4, depression T score 54.7)  “I did see the hospital psychologist once or twice…” (Participant 5, 22-25 years old and completed treatment, Acute lymphoblastic leukemia, anxiety T score 57.5, depression T score 50.8)  “… I saw [the psychologist] early on in my treatment… And he did say that I could use some of my hours, like, during treatment as clinical experience hours, which was nice.” (Participant 9, 15-21 years old and completed treatment, Acute lymphoblastic leukemia, anxiety T score 51, depression T score 37.1) |
| **Subtheme 3c:** Some participants leaned towards seeking professional help after reflecting back on their cancer experience. | “Therapist is something that had come up… with my doctor after my initial diagnosis… at that point I don’t know if it—where I was at the time, it wasn’t something that I thought I needed…as the years have gone by, maybe… I've considered… trying to re-initiate that sort of conversation, as it might be useful.” (Participant 30, 22-25 years old and completed treatment, Testicular cancer, anxiety T score 46.4, depression T score 46)  “And I feel like [talking to a psychologist] would have been beneficial for me to do so at the time, especially. Even now, I think about sometimes getting a therapist. But I just honestly don't have much time to look into it. But I just think I'd be interested just to like, talk to someone, talk to [someone] about outlooks on life, and my thoughts, and stuff like that.” (Participant 37, 15-21 years old and completed treatment, Ewing sarcoma, anxiety T score 61.5, depression T score 54.5)  “It was more after, like, a year in, I thought, maybe, that was something that— [mental health care] would be helpful.” (Participant 1, 22-25 years old and completed treatment, Acute lymphoblastic leukemia, anxiety T score 64.6, depression T score 43.1)  “I would say, like, people need to focus, definitely during this time, on their mental health because, like I said, it was a roller coaster for me. It wasn't the easiest. I would say I would kind of put this in as one of the hardest times of my life, 'cause of, you know, mental health, specifically… two months before I found out I had cancer, I had COVID…But yeah, I would say in terms of, like, the question and everything, like, the mental health questions are most important to anybody you would probably talk to, because I know they-- they're going through a lot.” (Participant 23, 22-25 years old and receiving treatment, Hodgkin lymphoma, anxiety T score 47.3, depression T score 48) |
| **Theme 4:** **Social isolation and loneliness were noted as consequences of physical isolation related to treatment, impacting psychological health.** | |
| **Subtheme 4a:** Participants experienced frustration at physical cancer treatment side effects, which impacted social well-being and psychological health. | “I became quite comfortable with [losing my hair] after a while. At first, it was really terrifying because I had super long hair. And then I chopped it before it fell out and then I shave it. And I was like, oh my God, I need to get a wig. I will not look pretty without hair. And then I [gave] up on that real quick when I realized how uncomfortable it was.” (Participant 13, 15-21 years old and completed treatment, Hodgkin lymphoma, anxiety T score 54.3, depression T score 48.3)  “… I imagine like in the next coming couple of months, or weeks, or whatever, once I actually get all my strength and stuff back… it's a slow process, I guess. It doesn't happen instantly, which is what I kind of-- it was hard to grasp. I just want it to happen, like, like that, so I could just not have to worry about anything…” (Participant 41, 15-21 years old and receiving treatment, Acute lymphoblastic leukemia, anxiety T score 61.4, depression T score 54.7)  “[Not feeling like myself] put me down. I think that's where I would get my most down moments and I needed the pick me uppers from my providers and family. Because I would get so happy because I'd finally not be throwing up anymore or not feeling weak. And I was able to run around, do… normal things I would do…And then it would just go right out the door. I feel like you had that little grasp of normalcy, and then it goes away. It kind of hurt a little bit because I was like, dang, it must be nice to feel well all the time. It's something you take for granted every day.” (Participant 13, 15-21 years old and completed treatment, Hodgkin lymphoma, anxiety T score 54.3, depression T score 48.3)  “And just… just like, not, just, like, slowly becoming weaker and I've been able to, like, lead a normal, healthy lifestyle. And it really, like, impacted me, like, emotionally very negatively…but it really, like, impacted negatively due to the fact that I [could not] function as I did before. And that was, like, a change that… I had [to] adapt to, that I didn't want to.” (Participant 34, 22-25 years old and completed treatment, Peripheral T cell lymphoma, anxiety T score 51, depression T score 37.1) |
| **Subtheme 4b:** Some experienced social isolation, largely from being physically alone during treatment. | “It did suck when COVID happened and then [my mother] wasn't allowed in with me because I was 18 at the time. That one hit a little hard because I think it was my first radiation treatment and I had to go in alone.” (Participant 13, 15-21 years old and completed treatment, Hodgkin lymphoma, anxiety T score 54.3, depression T score 48.3)  “So I couldn't have any friends or other family come and sit with me and visit for a little bit, since there were like no visitors allowed at the time, which was hard, for sure… I couldn't really talk to anyone and like kind of make the time go faster, say, when I was getting my treatment… I would kind of just have to sit there and just be on my phone and stuff. And I would FaceTime my friends a little bit. But it's obviously different without having someone like there with you. I mean, I did have my mom, obviously, which was very helpful.” (Participant 21, 15-21 years old and completed treatment, Hodgkin lymphoma, anxiety T score 58.2, depression T score 44.5)  “So obviously, I wasn't able to meet my friends that much out of school.” (Participant 37, 15-21 years old and completed treatment, Ewing sarcoma, anxiety T score 61.5, depression T score 54.5)  “In reality, as much as I was pushing through and how resilient I was, there was moments where, like, I did feel helpless and scared and alone and stuff like that.” (Participant 13, 15-21 years old and completed treatment, Hodgkin lymphoma, anxiety T score 54.3, depression T score 48.3)  “But I think… it was just lonely. And I think the world for the past two years, everyone's kind of felt that… regardless of whatever you're going through.” (Participant 36, 22-25 years old and completed treatment, Hodgkin lymphoma, anxiety T score 58.2, depression T score 46)  “I mean, it's isolation. It's isolating, but like just in terms of mental health, it's really hard to not even be-- a room that's now your own room. I think that I wasn't prepared necessarily for how like just kind of like on my own it would feel or like surreal, even.” (Participant 5, 22-25 years old and completed treatment, Acute lymphoblastic leukemia, anxiety T score 57.5, depression T score 50.8)  “I got none of my senior year. I got none. I was so disappointed. I didn't even get a glimpse of it. It was so disappointing.” (Participant 13, 15-21 years old and completed treatment, Hodgkin lymphoma, anxiety T score 54.3, depression T score 48.3) |
| **Theme 5:** **Despite experiencing negative emotions during their cancer journey, participants also described resilience and optimism.** | |
|  | “But I would say that, like, learning to take better care of myself was probably the biggest thing that came from it that was any sort of positive.” (Participant 40, 15-21 years old and completed treatment, Hodgkin lymphoma, anxiety T score 54.6, depression T score 48.3)  “… it sounds strange, but I am happier, in a way, because it feels like I actually have some sort of direction I'm working towards now. Like, it feels like I-- I understand that my time on this Earth is limited. And now that I understand that, I feel like what I am doing does have a little bit more purpose, even though I don't know what that purpose is. So don't get me wrong, if I could go back in time and never get diagnosed, I absolutely would take that decision. But… there are a little, little positive bits sprinkled throughout my diagnosis that keeps me moving forward.” (Participant 22, 15-21 years old and receiving treatment, Acute lymphoblastic leukemia, anxiety T score 67.8, depression T score 59.5)  “… but, I would say, like, there's so many ups and downs that, like, the ups are enough to, like, distract you from the downs.” (Participant 24, 15-21 years old and receiving treatment, Hodgkin lymphoma, anxiety T score 62.7, depression T score 58)  “… if like things that were occupying my mind during that time, like, I guess I felt isolated, but I wasn't too concerned about that, because I thought, you know, this is temporary, and those feelings will go away once I am back… in more of a normal situation.” (Participant 1, 22-25 years old and completed treatment, Acute lymphoblastic leukemia, anxiety T score 64.6, depression T score 43.1)  “Like most people that got diagnosed, like why me, why, why was I the one, why was I the one that had to get-- go through this? Like-- and there was, I mean, there was never a time where I was like, oh, I just want to give up. I don't do this anymore. I mean, obviously, there [were] times where I said I don't want to do this anymore. But more so meant go through it, but like I knew I needed to in order to live. So like I pushed through it and it taught me a lot about myself, definitely.” (Participant 19, 15-21 years old and completed treatment, Acute lymphoblastic leukemia, anxiety T score 59.7, depression T score 46.6)  “But… I would say that throughout my battle, I was honestly pretty upbeat. I never really had like seriously long bouts of depression.” (Participant 36, 22-25 years old and completed treatment, Hodgkin lymphoma, anxiety T score 58.2, depression T score 46)  “I mean, I'm thankful for the advances we've made over the years, honestly, because I would not be alive if we didn't. So that's really cool… but it is stressful. You know, you don't know exactly what the outcome is going to be, so it's stressful, especially at the beginning. You know, once you start seeing numbers move, you kind of get used to them real quick. At first, it was really stressful. But overall, it's just… it's just something you go with, really.” (Participant 4, 15-21 years old and receiving treatment, Chronic myeloid leukemia, anxiety T score 59.4, depression T score 43.1)  “But I think [the experience with cancer treatment] just helped bring out, like, a positive side of me that… wasn't there.” (Participant 12, 15-21 years old and receiving treatment, Hodgkin lymphoma, anxiety T score 60.3, depression T score 56.3)  “I mean, I don't really know how to explain it, to be honest. It just feels-- I mean, [cancer is] a part of, I guess, my journey, quote, unquote, I guess you can say. But I don't mind talking about it. It doesn't really get me upset or anything like that.” (Participant 2, 22-25 years old and completed treatment, Hodgkin lymphoma, anxiety T score 48.3, depression T score 37.1)  “And… you, you always need to think, you never, you never have, you never have the worst. There's always somebody out there who's, who's doing worse than you… but that's, you know, something that I always, always… reflect on. There's, you know, there's somebody out there who's, who's got [it] worse. And you just got to, those are the cards you're dealt and play 'em.” (Participant 27, 22-25 years old and completed treatment, Hodgkin lymphoma, anxiety T score 49.4, depression T score 52.5)  “… if one day I, you know, have a relapse or whatever may happen, then there's a time for that. But right now, I'm like very… I feel like I can see the world in full color.” (Participant 36, 22-25 years old and completed treatment, Hodgkin lymphoma, anxiety T score 58.2, depression T score 46) |
